# Fish-Based Biopolymer Complex Coacervate Coating for Improved Paper Oxygen and Water Barrier

**DOI:** 10.1021/acs.biomac.5c02091

**Published:** 2025-11-26

**Authors:** Sarah G. Fisher, Zachary Buck, Margaret J. Karim, Jaime C. Grunlan

**Affiliations:** 14736 Texas A&M University, 400 Bizzell Street, College Station, Texas 77840, United States

## Abstract

Food packaging is critical to prevent food waste, but most of the packaging used today is not sustainable. Paper-based packaging materials offer a renewable option, but exhibit poor resistance to common permeants such as oxygen, grease, and water vapor. In this work, a complex coacervate coating is prepared from two waste biopolymers, gelatin and DNA, and applied to kraft paper to substantially improve its barrier properties. Thermally curing the coating after deposition decreases the water vapor transmission rate and oxygen transmission rate by 83 and 99%, respectively, relative to uncoated paper. This work represents one of the best fully biobased barrier coatings reported for paper and is a promising option for sustainable food packaging.

## Graphical abstract

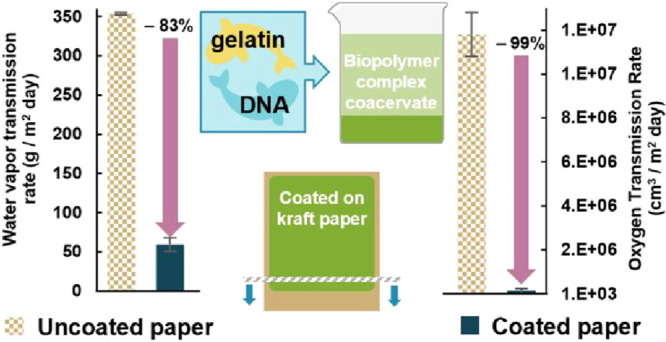

## Introduction

On average, about 30% of the food produced globally is lost or wasted before it can be consumed.^1^ Packaging is required to extend the shelf life of food, increasing its chance of being eaten instead of wasted.^2,3^ Two of the key permeants contributing to food degradation and decreased shelf life are oxygen and water vapor. Exposure to oxygen and/or moisture can promote microbial growth, lipid oxidation, decreases in nutritional value, and undesirable changes in food color, flavor, and texture.^2,3^ In addition, oily food requires packaging that is grease resistant.^4^ Increasing global awareness regarding sustainability has driven a strong interest in the development of renewable packaging options that retain the essential barrier properties required for food.^2,3,5–8^

One such sustainable packaging option is paper and paperboard. Paper is made of networks of interlaced cellulose fibers and has been utilized for food packaging since the 17th century.^7,9,10^ Paper-based packaging materials are primarily desirable for their lightweight and printability. Additionally, since they are biobased, biodegradable, home compostable, and recyclable, they have a lower environmental impact compared to other traditional packaging materials.^3,7,8,11^ Despite its benefits, untreated paper is not sufficient to protect food from oxygen, water, or grease due to its extremely high porosity and superhydrophilicity.^2,7,9,11^ Coatings are almost always applied to paper-based food packaging materials to improve their barrier performance, typically consisting of synthetic, petroleum-based polymers like polyethylene or poly­(ethylene terephthalate), or metals like aluminum.^7,9,11^ While coatings substantially improve the barrier performance of paper for applications in food packaging, they also negate sustainability by impeding biodegradability and recyclability.^7,11^ For this reason, there is great interest in utilizing bioderived polymers for paper barrier coatings. Biopolymers are generally nontoxic, readily available, sustainably sourced, biodegradable, and compostable.^7,8,11–14^ With that said, many biopolymers are inherently hydrophilic and do not provide good barrier performance alone due to their water sensitivity.^7,8^

The barrier performance of biopolymer coatings can be significantly improved by exploiting the electrostatic interactions that occur between charged biopolymers to form a biopolyelectrolyte complex. Polyelectrolyte complexes consisting of two or more oppositely charged polyelectrolytes, including biopolyelectrolytes, have demonstrated exceptional oxygen barrier performance and reduced water sensitivity due to the high cohesive energy density resulting from the many ionic cross-linking sites between the oppositely charged polymers.^15–18^ One form of polyelectrolyte complex, which occurs naturally in animals, like sandcastle worms and mussels, is a complex coacervate.^19,20^ Complex coacervation is a liquid–liquid phase separation initiated by electrostatic and other attractive interpolymer interactions, leading to the association of oppositely charged polymers in a viscous coacervate phase. This associative behavior is driven by the entropic gain of released counterions and water into the polymer-poor phase, called the supernatant.^19–21^ Biopolymers such as DNA and gelatin readily form complex coacervates under the correct conditions.^22–25^ Macroscopic phase separation of complex coacervates yields a viscoelastic polymer-rich phase (the coacervate phase) that can be applied as a coating for improved gas barrier or a range of other functionalities.^26–30^ Complex coacervates have also been used in the microencapsulation of various oils, suggesting their potential to act as a barrier to oil and grease in addition to oxygen and water vapor.^31–34^

In this work, a fully biobased complex coacervate is prepared utilizing fish gelatin and DNA, both of which are considered waste byproducts of the fishing industry.^35,36^ The polymer-rich coacervate phase is applied as a barrier coating on kraft paper in a single step. The coating alone demonstrates a 70% reduction in the oxygen transmission rate, a 29% improvement in the water vapor transmission rate, and a substantial improvement in the kit grease resistance of uncoated paper at a relatively low coat weight. Subsequent thermal treatment at 150 °C further decreases the oxygen and water vapor transmission rates by 99 and 83% relative to uncoated paper, respectively. This work is believed to represent the first fully biobased complex coacervate barrier coating applied to paper for sustainable food packaging.

## Materials and Methods

### Materials

Gelatin from cold-water fish skin, DNA from herring sperm (<50 bp, degraded), hydrochloric acid (HCl, ACS reagent, 37%), and sodium hydroxide (NaOH, ACS reagent, pellets) were purchased from Sigma-Aldrich (Milwaukee, WI, USA). DNA was stored at 7 °C until use. Kraft builder’s paper (TRIMACO Easy Mask, 10 mil, 106 g/m^2^) was purchased from Home Depot (College Station, TX, USA) and used as the substrate for coat weight, thickness, scanning electron microscopy, moisture content, water absorptivity, contact angle, water vapor transmission rate, oxygen transmission rate, and kit test experiments. Single-side polished, 500 μm-thick Si wafers were purchased from University Wafer (Boston, MA, USA) and used as substrates for FTIR, atomic force microscopy, and thermal degradation experiments.

### Complex Coacervate Preparation

Eighteen MΩ deionized (DI) water was used to prepare all solutions. DNA solutions were prepared by dissolving 13 wt % DNA in DI water and adjusting the pH to ∼3 with 5 M NaOH to promote solvation. After DNA was fully dissolved, the solution pH was adjusted to 2.3 with 5 M HCl. Gelatin solutions were prepared by dissolving 13 wt % gelatin in DI water and adjusting the pH to 2 with 5 M HCl. Equal volumes of gelatin and DNA solutions were combined and stirred for 1 min, then allowed to equilibrate for 18 h to ensure stability. After 18 h, the less-dense supernatant phase was decanted and the coacervate phase was retained for coating.

### Coating Deposition

Immediately before coating, silicon wafer substrates were rinsed with DI water, methanol, DI water again, dried with compressed air, and plasma treated using a PDC-32G plasma treater (Harrick Plasma, Ithaca, NY, USA) for 5 min to improve coating adhesion. Paper was not pretreated. The coacervate coating was applied on one side of the substrate with a #10 wire-wound film applicator rod (Paul N. Gardner Company, Columbia, MD, USA), after which the substrate was allowed to dry flat at room temperature for 24 h. After drying, films were left uncured or thermally cured for 24 h at 105, 130, or 150 °C before the samples were slowly cooled back to room temperature. The coating process could easily be scaled up for industrial application using roll-to-roll processing techniques.

### Characterization

Zeta potential measurements were made in triplicate using a Malvern Zetasizer Nano ZS, with 0.1 wt % solutions of gelatin and DNA, at pH values ranging from 2 to 10. Solids content was determined by drying ∼5 g of coacervate or supernatant at 70 °C for 24 h and taking the mass before and after. An average of three measurements is reported. The viscosity and rheological properties of the coacervate and supernatant were measured using a TA Instruments DHR-2 Rheometer (New Castle, DE, USA). A flow sweep procedure from 1 to 1000 s^–1^ was performed using a 40 mm parallel plate attachment with a 500 μm gap. Because the coacervate exhibits some shear thinning behavior, the viscosity was calculated as the average of six viscosity measurements taken from 100 to 1000 s^–1^. FTIR spectroscopy was performed using an Alpha Platinum-ATR FTIR spectrometer (Bruker Optics Inc., Billerica, MA, USA), with air as the background. A minimum of 24 scans were signal averaged for each sample, and the scan resolution was 4 cm^–1^. DNA and gelatin were analyzed as received. Coacervates and supernatants were dried at 70 °C for 24 h to remove water, then ground with a mortar and pestle to yield a powder. Coatings were prepared as described above on Si wafers, then scraped off with a razor and ground with a mortar and pestle to yield a powder. X-ray photoelectron spectroscopy (XPS) was performed using an Omicron XPS/UPS (Uppsala, Sweden), with a DAR 400 Mg/Al X-ray source and a 0.8 eV energy resolution detector. Samples were prepared as described for FTIR.

Coating thickness was measured using a ProGage Touch Thickness Tester (Thwing-Albert Instrument Company, West Berlin, NJ). An average of 12 measurements was reported. Basis weight was measured by punching a 1” diameter circle of uncoated or coated paper, taking the mass with an analytical balance, and dividing by the area of the circle (in m^2^). An average of five measurements was reported. Scanning electron microscope (SEM) images of the surface of uncoated and coated paper were obtained using a Model JSM-7500F FE-SEM (JEOL, Tokyo, Japan) after sputter coating with 5 nm of palladium–platinum alloy.

Thermal degradation of coatings and components, as well as the moisture content of uncoated and coated paper samples, was determined using a Discovery TGA 55 (TA Instruments, New Castle, DE, USA). For thermal degradation experiments, samples were prepared as for FTIR. ∼10 mg samples were subjected to a ramp of 10 °C per minute from 120 to 800 °C under air. The 5% degradation temperature was determined to be the temperature at which 95% of the original mass at 120 °C remained, and an average of three measurements was reported. For moisture content experiments, 2–3 mg samples were subjected to a 30 min isotherm at 100 °C under air and the change in mass was measured. An average of three measurements was reported. Water absorptivity was measured by punching a 1” diameter circle of uncoated or coated paper and taking the mass with an analytical balance before and immediately after 10 min in a 94% relative humidity (RH) chamber. The moisture absorptivity was calculated as the percentage increase in the mass. An average of four measurements was reported.

Static water contact angles were measured using a KSV CAM 200 instrument (KSV Instruments, Ltd., Monroe, CT, USA). Data was recorded 30 s after depositing a 5 μL DI water droplet on the surface of coated or uncoated paper. An average of 10 data points (5 water droplets) was recorded for each sample. Water vapor transmission rate (WVTR) was measured in triplicate according to ASTM E96/E96M–24, using the water method, with the samples having the coated side up at 30 °C and an average of 50% RH. Each run lasted 3 h, and the region of the curve corresponding to the final hour of testing was used to calculate the water vapor transmission rate. Grease resistance was measured according to the TAPPI T559 “kit” test. An average of five measurements was reported. Oxygen transmission rate (OTR) was performed by MOCON Inc. (Minneapolis, MN, USA) using an OpTech-O2 model P instrument. OTR testing utilized room air (20.9% oxygen) as a test gas at 23.0 °C, with a relative humidity (RH) of 39%, in accordance with ASTM F3136. An average of two measurements was reported. Atomic force microscopy (AFM) was performed with a Bruker Dimension Icon AFM (Billerica, MA, USA). An average of the average roughness of three images was reported.

## Results and Discussion

### Coacervate Properties

Representative structures of complex coacervate components and the complex coacervate preparation procedure are shown in Figure 1a,b, respectively. Zeta potential values of DNA and gelatin at various pH values are shown in Figure S1. Gelatin and DNA solutions were combined at a pH of ∼2, where gelatin is cationic and DNA is anionic. Upon combination and agitation, the gelatin/DNA mixture spontaneously separates into two liquid phases: a viscous, polymer-rich coacervate phase and a waterlike, polymer-poor supernatant. The phase separation is driven by the entropic gain of released counterions and water, which outweighs the entropic loss of the larger polyelectrolytes as they coalesce, first into ionically associated droplets, and then into a single dense macrophase to reduce total interfacial area.^19–21^ While some macroscopically phase-separated complex coacervates (hereafter referred to simply as “coacervates”) require thermal annealing or aging for phase separation, biomaterials like DNA and gelatin readily form coacervates under ambient conditions.^22–25^ Macroscopic phase separation begins immediately after mixing and the mixture is allowed to stabilize for 18 h before use. After this period, the two liquid phases can be clearly distinguished and characterized.

**Figure 1:**
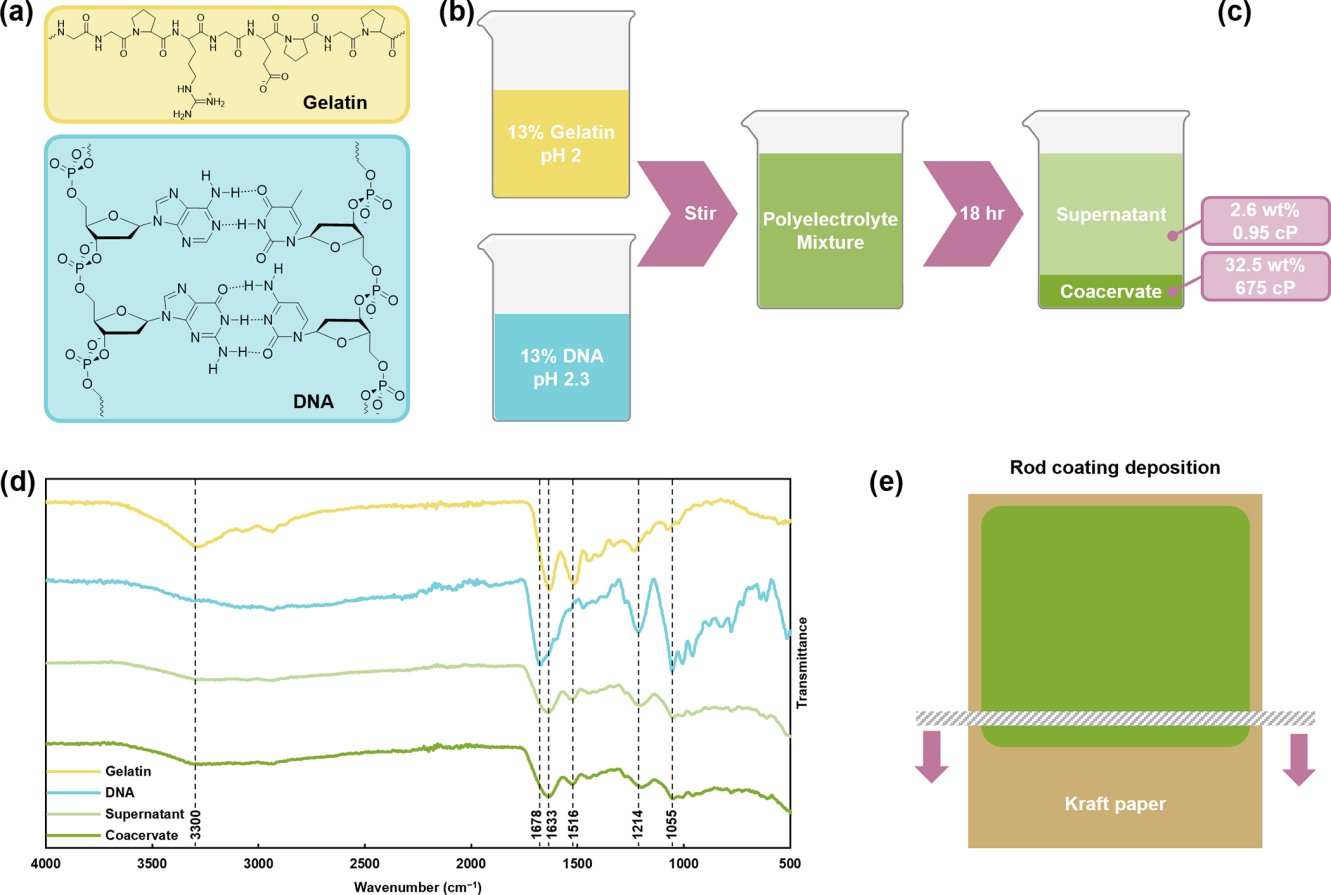
(a) Chemical structures of coacervate components, (b) preparation of coacervate, (c) solids content (in wt %) and viscosity (in centipoise) of supernatant and coacervate phases, (d) FTIR spectra of components, supernatant, and coacervate, and (e) deposition of coacervate coating. Created with BioRender.com.

As denoted in Figure 1c, the polymer-poor supernatant phase is 2.6 ± 0.4% solids by mass, with a viscosity of 0.95 ± 0.04 cP (1 cP = 1 mPa s), while the polymer-rich coacervate is substantially denser and more viscous at 32.5 ± 1.2% solids and 675 ± 8 cP. Viscosity as a function of shear rate is shown in Figure S2. Importantly, complex coacervation is an associative phase separation process, meaning both polyelectrolytes are expected to be present in the coacervate phase (and, to a lesser extent, the supernatant phase).^19–21^ FTIR confirms the presence of both polymers in both phases of the coacervate. The spectra of each coacervate component, and each phase of the coacervate, is shown in Figure 1d. The gelatin spectrum exhibits the key bands at 3300, 1633, and 1516 cm^–1^, which correspond to the amide A N–H stretch, amide I carbonyl stretch, and amide II N–H bend, respectively.^37^ Likewise, DNA exhibits characteristic peaks at 1678, 1214, and 1055 cm^–1^, attributed to the cytosine, guanine, and thymine carbonyl stretches, P=O and PO_2_^–^ stretching, and C–O–C and C–O–P vibrations, respectively.^38–40^ The FTIR spectra of the supernatant and coacervate show evidence of all of these characteristic peaks. The amide I, amide II, phosphate stretches, and C–O–C and C–O–P vibrations are all clearly apparent in both spectra, while the nitrogenous base carbonyl stretch appears as a new shoulder on the amide I peak. The amide A stretch at 3300 cm^–1^ is broader and weaker but is still evident. Therefore, FTIR data suggests that both components are found in the coacervate, and to a lesser extent (due to the significantly reduced concentration) in the supernatant.

The composition of the two phases is further confirmed via XPS. As shown in Table S1, gelatin has a phosphorus to nitrogen (P:N) ratio of 0, as there is no detectable phosphorus in gelatin. DNA, on the other hand, contains significant amounts of both phosphorus and nitrogen and has a P:N ratio of 0.41. The coacervate and supernatant phases exhibit P:N ratios of 0.13 and 0.31, respectively. Because both of these values fall between the P:N ratios for pure gelatin and pure DNA, it is confirmed that both phases contain a mixture of the two polymeric components. An equimolar coacervate phase is expected to correspond to a P:N ratio of ∼0.17 (Table S2). It is believed that the supernatant phase contains excess DNA that was excluded from the coacervate due to the greater mobility of the relatively flexible gelatin polymer backbone as opposed to the very rigid DNA structure.^41^ It is also possible that excess gelatin is present in the coacervate due to a mismatch in charge density between DNA and gelatin at pH 2. According to the zeta potential data presented in Figure S1, DNA carries a greater magnitude of charge (−20.0 ± 0.9 mV) than gelatin (+15.6 ± 3.2 mV) at pH 2, so a slight excess of gelatin may be necessary for charge compensation in the coacervate phase. The high concentration of sodium detected in the supernatant phase is also to be expected, as small counterions are expelled from the coacervate phase into the supernatant phase during coacervation. It is important to note that most of this sodium is expected to originate from the sodium hydroxide used to help dissolve the DNA, leading to a higher concentration of sodium in the supernatant than in either DNA or gelatin.

### Coating Deposition and Curing

Upon phase separation and equilibration, the supernatant phase was discarded and the coacervate was coated onto kraft paper as depicted in Figure 1e. After air drying, the coatings were subjected to a 24-h thermal cure at 105, 130, or 150 °C. Some coatings were also left uncured for comparison. FTIR spectra of the uncured and cured coacervates (Figure S3) indicate minimal changes in the chemical structure of the coating upon curing. This indicates that the “curing” effect is likely primarily physical.

Polyelectrolyte complex systems have been reported to undergo restructuring upon thermal annealing. For example, Köhler and co-workers prepared polyelectrolyte multiplayer microcapsules of poly­(diallyldimethylammonium chloride) (PDADMAC) and poly­(styrenesulfonate) (PSS) and found that thermally annealing at 120 °C provided the thermal energy necessary to allow irreversible polyelectrolyte rearrangement (and potentially the expulsion of bound water) to a more thermodynamically stable state.^42,43^ A similar observation was made for polyelectrolyte multilayers of poly-l-lysine and alginate, where thermal annealing resulted in smoother, stiffer, and more hydrophobic films via densification and restructuring.^44^ Nanofibers of chitosan and gelatin were also found to increase in strength and decrease in swelling capacity upon curing at temperatures from 60 to 150 °C. Again, the change in properties due to annealing was attributed to intensified intermolecular interactions promoted by thermal treatment.^45^ It is believed that the curing mechanism of the gelatin/DNA coatings reported herein functions similarly, with the input of thermal energy promoting the restructuring and densification of the coacervate coatings to maximize favorable interpolymer interactions, with minimal chemical transformation. The curing temperatures selected are below the 5% degradation temperature of DNA, gelatin, and the coacervate, as indicated in Figure S4, so it is not expected that significant thermal degradation occurs during the curing process. The curing times could likely be shortened for more practical industrial application.

As shown in Figure 2a, the coatings increase the thickness of the paper substrate from 169 μm for uncoated paper to 180–201 μm. There is no apparent thickness trend with curing. Similarly, the basis weight (mass as a function of area) of coated paper increases from 111 g/m^2^ to 136–143 g/m^2^, a 23–29% increase in basis weight, with no apparent thickness trend between coatings (Figure 2b). This weight gain is on the lower end of reported coacervate coatings on paper.^28,30^ The high variability in thickness and basis weight is attributed to the heterogeneous nature of paper. The roughness of the coatings is also unchanged with curing (Figure S5).

**Figure 2:**
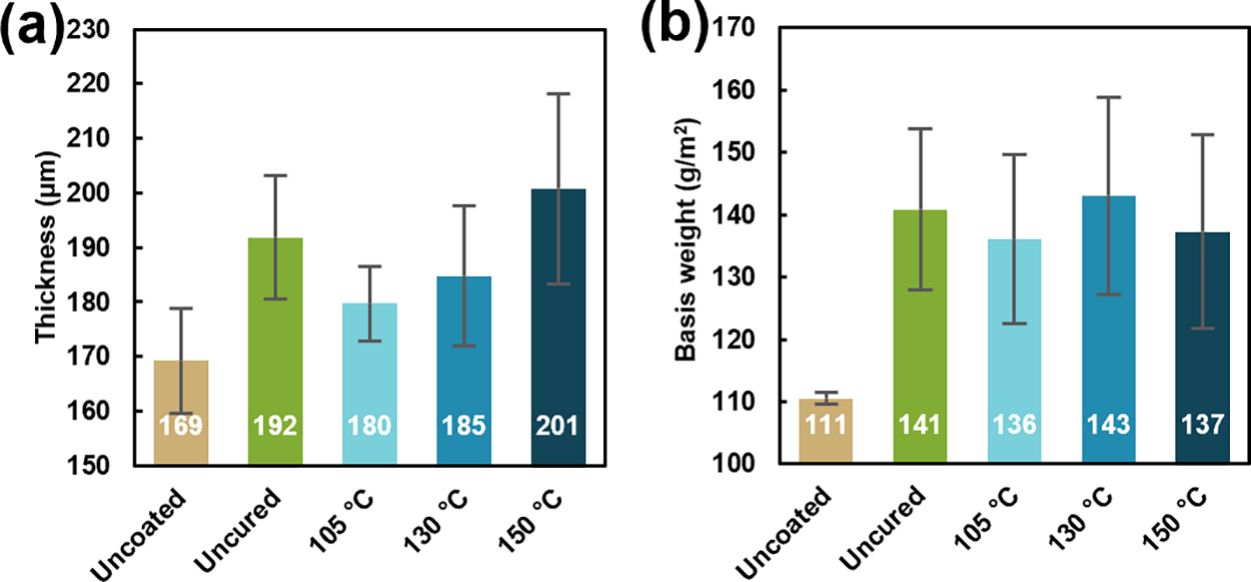
(a) Thickness and (b) basis weight of uncoated and coated paper.

SEM images reveal the morphology of uncoated and coated paper. As seen in Figure 3a,f, uncoated paper is rough and porous, which results in extremely poor barrier performance. Upon coating, the paper fibers are still somewhat visible, but the large pores and spaces between fibers are filled in with the coating, resulting in a smoothing effect (Figure 3b–e). Upon closer inspection, the coatings exhibit large cracks scaling hundreds of microns (Figure 3g–i). These cracks are expected to act as a “highway” for permeants, preventing effective barrier performance of the coatings. The cracks are most prevalent in the uncured and 105 °C cured coatings (Figure 3g,h, respectively). When cured at 130 °C, the cracks are shorter and appear less deep, as shown in Figure 3i. After curing at 150 °C (Figure 3j), no cracks are apparent on the surface of the coating. The decrease in cracking behavior at higher curing temperatures is believed to occur due to the previously discussed restructuring of the coacervate coating after deposition, relieving internal stresses to create a tougher coating less prone to cracks.^42–45^

**Figure 3:**
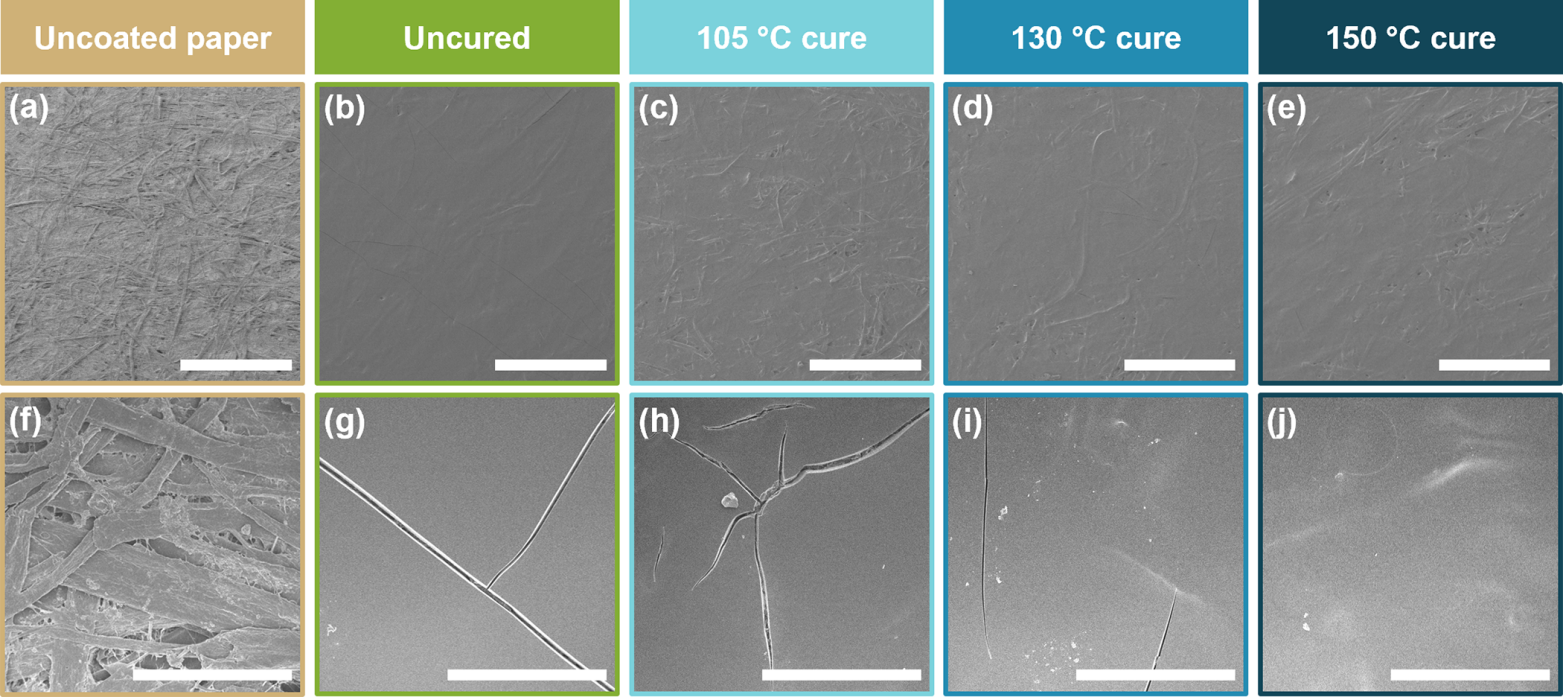
SEM images of (a, f) uncoated paper and coated paper with varying curing temperatures: (b, g) uncured, (c, h), cured at 105 °C, (d, i) cured at 130 °C, and (e, j) cured at 150 °C. Scale bars are (a–e) 1 mm and (f–j) 100 μm.

### Water Barrier of Coated Paper

Biopolyelectrolytes are extremely hydrophilic and can result in increased water sensitivity when applied as a coating.^46,47^ For this reason, the ambient moisture content and water absorptivity of uncoated and coated paper was investigated. As shown in Figure 4a, uncoated and coated paper show similar moisture content values between 7 and 10% by mass. These values are comparable to previously published coacervate coatings on paper.^30^ The uncured coating slightly increases the moisture content of uncoated paper from 8.2 to 9.4, but curing decreases the moisture content to a comparable or slightly lower value than uncoated paper. The water uptake of uncoated and coated paper was also measured under extremely humid conditions (94% RH) to determine water absorptivity by mass. As shown in Figure 4b, the presence of the coating does not increase the water absorption of paper in a humid environment. Generally, increasing the cure temperature of the coating decreases the water absorptivity under high humidity, indicating the role of the curing process in strengthening intermolecular interactions to provide improved water resistivity.^42–45^

**Figure 4:**
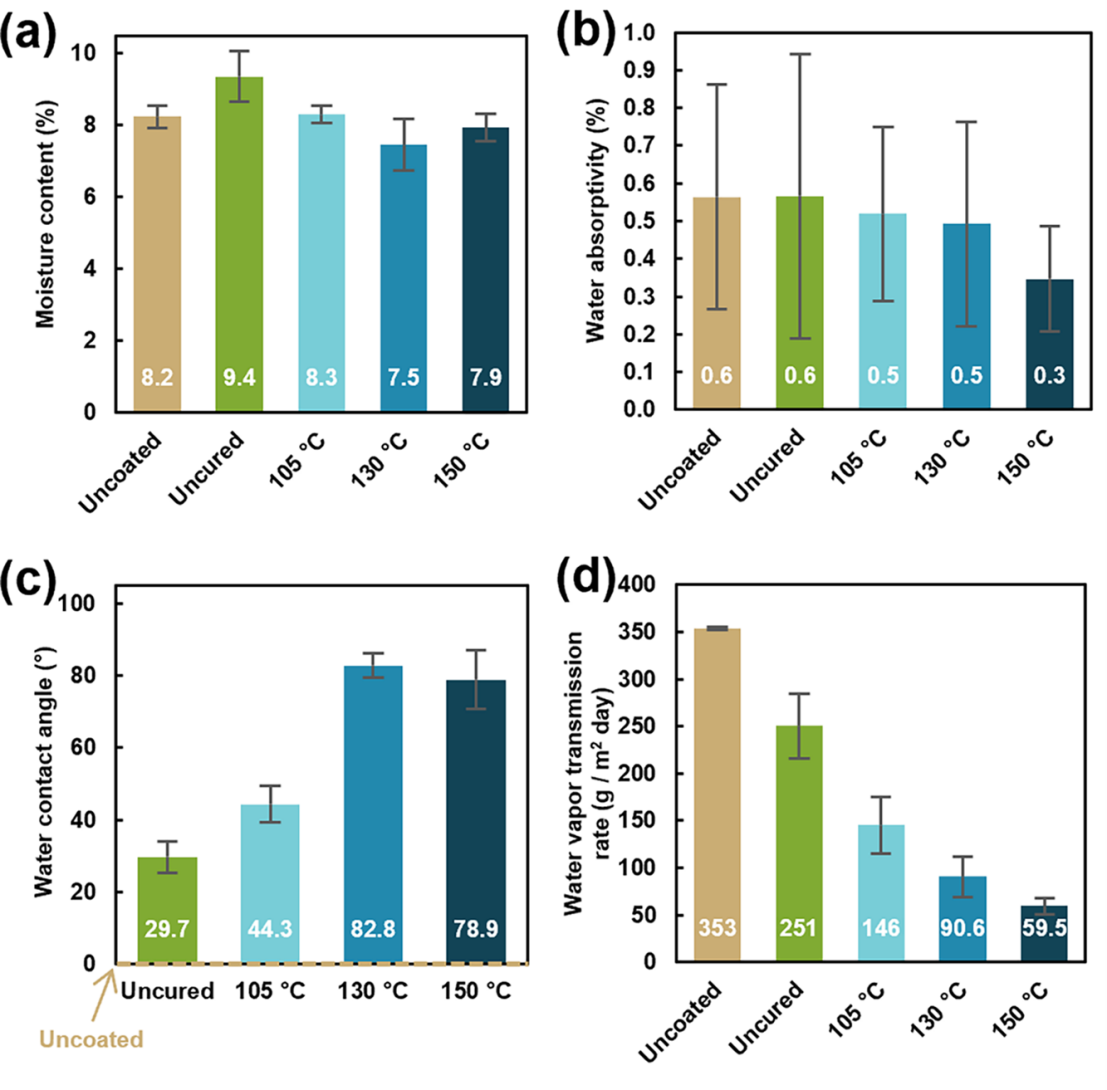
(a) Moisture content, (b) water absorptivity, (c) water contact angle, and (d) water vapor transmission rate of uncoated and coated paper. Uncoated paper has a contact angle of 0, as denoted by the dotted line in (c).

This increase in water resistivity with curing is even more apparent when examining the water contact angle of uncoated and coated paper, as shown in Figure 4c. Uncoated paper absorbs water upon contact, leading to a contact angle of 0. Upon coating with the coacervate, the contact angle is increased to 30°. Thermally curing at 105 °C further increases the contact angle to 44°, while curing at higher temperatures of 130 or 150 °C results in a contact angle of ∼80°. This value is comparable to other contact angles reported for coated papers for food packaging.^48^ It is clear that the thermal curing process and subsequent coacervate restructuring significantly improve the ability of the coating to resist water.

This water resistance is also observed in water vapor transmission rate measurements, as shown in Figure 4d. Uncoated paper exhibits a WVTR of 353 g/m^2^ day. As previously mentioned, paper is a poor barrier to water vapor due to its hydrophilicity and extreme porosity. All of the coatings dramatically improve the WVTR of uncoated paper by filling in the pores and reducing the hydrophilicity. However, the curing process and temperature are critical to achieving the best barrier performance. While the uncured coating reduces the WVTR of paper by ∼100 g/m^2^ day, curing at 105 °C reduces WVTR by over 200 g/m^2^ day. Curing at 130 and 150 °C further reduces the WVTR to 90.6 and 59.5 g/m^2^ day, respectively. The best-performing coating, cured at 150 °C, performs ∼6× better than uncoated paper and ∼4× better than uncured coated paper. This highlights the substantial impact of thermal curing on water barrier performance.

The water vapor barrier improvement achieved is a significant result for a fully biobased paper coating, especially when compared with other results from the literature. Table 1 summarizes key barrier results from recently published studies examining biobased coatings on paper and paperboard substrates. Due to differences in substrates’ inherent barrier, the relative barrier improvement is reported. The biopolymer coacervate coating reported in this work outperforms many other biobased barrier coatings in reducing the water vapor transmission of the substrate. A few other recently reported biobased barrier coatings achieve a greater improvement in WVTR, namely poly­(lactic acid) [PLA] and PLA with potato extract and poly­(butylene adipate terephthalate) [PBAT].^49,50^ However, both of these coatings require substantially higher coat weight to achieve this barrier improvement. Additionally, while PLA is synthesized from monomers derived from biomass, it requires a multistep synthetic pathway to produce and, in contrast to natural biopolymers, it does not readily degrade in the natural environment or under home composting conditions.^51–53^

**Table 1:** Coat Weight and Water Vapor Transmission Rate Improvement of Biobased Barrier Coatings on Paper and Paperboard Substrates^a^

biobased coating	coat weight (g/m^2^)	WVTR improvement	reference
gelatin/DNA	26	5.9×	this work
chitosan (CH)/pectin/carnauba wax	2.4	1.3×	^54^
poly(lactic acid) [PLA]	8.3	5.1×	^55^
	81	10.9×	^49^
PLA/potato extract/poly(butylene adipate terephthalate) [ PBAT]	329	26.7×	^50^
PLA/polycaprolactone	3.7	1.4×	^56^
cellulose nanofibers	26	4.1×	^57^
cellulose undecenoyl ester	21.4	3.5×	^58^
CH/microcrystalline cellulose/protein	7.1	3.2×	^59^
nanocellulose/citric acid	61	1.4×	^60^
CH	6	3.2×	^61^
	2.3	1.1×	^62^
alginate	6.1	1.8×	^61^
starch	8	1.1×	^63^
poly(vinyl alcohol)/zein	∼67	2.9×	^64^
poly(dimethylsiloxane)/zein	7.2	3.3×	^65^

a Relative improvement is reported to account for differences in paper substrates utilized.

### Oxygen and Grease Barrier of Coated Paper

Oxygen barrier performance is less commonly reported than water vapor transmission, but it is a critical property for food packaging materials in order to prevent spoilage.^2,3^ As shown in Figure 5, the rate of oxygen transmission through uncoated paper is almost 12 million cm^3^/m^2^ day. Applying the coacervate coating with no curing improves the oxygen transmission rate by a factor of ∼6.4. As with water vapor barrier, further improvements in oxygen barrier are observed with thermal curing, with the highest cure temperature of 150 °C resulting in the lowest OTR value of 128,000 (a 92× or 99% improvement). This substantial improvement in barrier performance is attributed to an increase in interpolymer interactions and improved polyelectrolyte packing that are promoted by thermal treatment, leading to less free volume within the film for penetrants to occupy. The OTR attained by the 150 °C cured coating is comparable to one previously reported with a nonbiobased coacervate coating,^30^ and is accomplished with substantially lower weight gain and the added benefit of increased sustainability. It is important to note that while the error associated with the OTR data is relatively high, this is primarily attributed to the heterogeneity of the paper substrate itself. While uncoated paper deviates by almost 1,000,000 cm^3^/m^2^ day and uncured coated paper has a high standard deviation of >2,000,000 cm^3^/m^2^ day, all the coated and cured paper samples show significantly lower standard deviations (on the order of 300,000 cm^3^/m^2^ day). These values are high relative to the average OTR reported for coated paper, but are understood to be caused by the substrate, which shows massive variation in barrier properties between individual samples.

**Figure 5:**
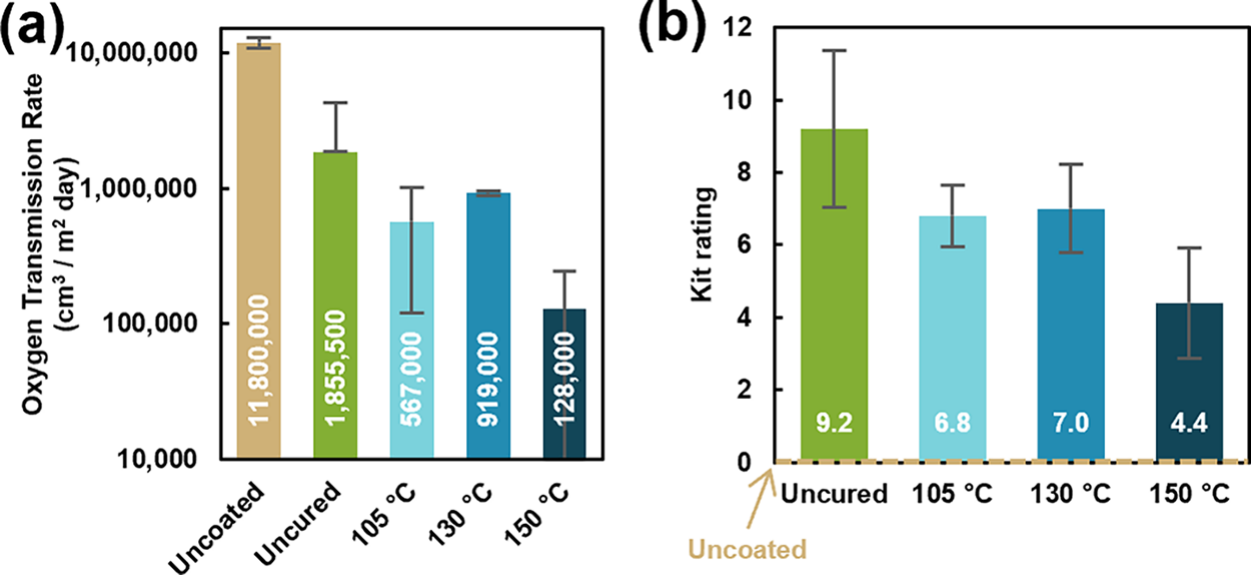
(a) Oxygen transmission rate and (b) Kit ratings of uncoated and coated paper. Uncoated paper has a Kit rating of 0 (unrated), as denoted by the dotted line in (b).

The WVTR and OTR behavior achieved by coated paper cured at 150 °C makes it an appropriate material for packaging fruits, vegetables, and salads.^66^ Few biobased barrier coatings for paper achieve a similarly effective combination of water vapor barrier and oxygen barrier. Table 2 shows the relative barrier improvement and the coat weight of paper-based materials coated with biobased barrier coatings. Some literature reports only gas transmission rate, while others report only gas permeability, so both values are reported for the gelatin/DNA coacervate coating to ensure accurate comparison. Of the literature that reports both water vapor and oxygen barrier performance, only this work demonstrates an 80+ % improvement in both parameters simultaneously. A natural rubber-based coating developed by Adibi et al. provides comparable WVTR improvement and even better OTR improvement, but the coat weight is not reported, and the coating thickness is 100 μm (over 3× thicker than the coatings presented in this work).^67^ As a result, it is likely that the coat weight is substantially higher than that of the coatings reported in this work.

**Table 2:** Coat Weight, WVTR Improvement, Water Vapor Permeability (WVP) Improvement, OTR Improvement, and Oxygen Permeability (OP) Improvement of Biobased Coatings Applied to Paper^a^

biobased coating	coat weight (g/m^2^)	WVTR improvement	WVP improvement	OTR improvement	OP improvement	reference
gelatin/DNA	26	83.1%	80.0%	98.9%	98.7%	this work
NR/A-1,3G			78.9%		99.7%	^67^
CH/PT/CW	2.4	24.6%		95.1%		^54^
NC/CA	61	26.1%			99.5%	^60^
starch	8	12.1%		97.5%		^63^
PLA	8.3	80.5%		51.7%		^55^

a NR, natural rubber; A-1,3G, alpha-1,3 glucan; CH, chitosan; PT, pectin; CW, carnauba wax; NC, nanocellulose; CA, citric acid; and PLA, poly­(lactic acid).^54,55,60,63,67^

Grease resistance is a key requirement for packaging greasy or oily foods. It is commonly measured using the kit (TAPPI T559) test, where a higher rating on a scale of 1 to 12 indicates better resistance to grease.^61,68^ A kit rating of 6 or higher is required for most food packaging applications.^48^ Uncoated paper is extremely susceptible to grease due to its high porosity.^48,61,69^ As a result, uncoated paper is unrated in the kit grease resistance test (Figure 5b). All of the coated papers exhibit a substantial improvement in grease barrier, which is likely explained primarily by the physical effect of smoothing the surface and reducing the porosity of the paper.^70^ Coated, uncured paper achieves a kit rating of 9.2. Curing results in a decreased kit rating of ∼7, which continues to drop as cure temperature is increased, with the 150 °C cured coating achieving a rating of 4.4. The decrease in kit rating as cure temperature increases may be caused by a decrease in hydrophilicity upon thermal restructuring, as most (or all) charges in the film are expected to be intrinsically compensated. All of the coated papers except the 150 °C cured sample exhibit acceptable kit values for paper packaging applications.^48^

DNA and gelatin are known to biodegrade under mild conditions.^71–73^ As a result, the coated paper substrates are expected to maintain their desirable attributes of biodegradability and compostability. Paper coated with a biopolyelectrolyte complex is also expected to maintain its mechanical properties, as shown in a previous study.^54^ Polyelectrolyte complex films are known to demonstrate good long-term stability,^74,75^ but can be sensitive to high humidities.^76–78^ Based on the observed water resistance, cured coacervate coatings are not expected to be susceptible to performance loss in humid environments. Future work could entail confirming biodegradation via soil burial experiments, testing the mechanical durability, and confirming the long-term stability of the coacervate coatings.

## Conclusions

Two fishing industry waste products, gelatin and DNA, were applied as a complex coacervate barrier coating on kraft paper for use in sustainable food packaging. The coating is biobased, nontoxic, renewably sourced, and expected to be both biodegradable and compostable. Coated paper was cured at several temperatures, promoting internal restructuring and strengthened interpolymer interactions. The thermal treatment decreases moisture sensitivity and substantially improves water vapor barrier and oxygen barrier properties, which are essential for effective food packaging. The combination of oxygen barrier and water vapor barrier achieved is among the best reported for fully biobased paper coatings, and grease resistance was also improved. This work is a promising result for the future of renewable food packaging.

## Supplementary Material



## Notes

Zeta potential of DNA and gelatin across a pH spectrum (Figure S1); rheology of supernatant and coacervate (Figure S2); FTIR spectra of uncured and cured coatings (Figure S3); thermal degradation curves and 5% degradation temperatures of coacervate and components (Figure S4); average roughness and AFM images of coacervate coatings on Si waters (Figure S5); XPS data for coacervate components, coacervate, and supernatant (Table S1); and calculated Gelatin:DNA ratios from XPS data (Table S2) (PDF)
