# Supplementary material for: Fish-Based Biopolymer Complex Coacervate Coating for Improved Paper Oxygen and Water Barrier
Source: Biomacromolecules. 2025 Nov 26;27(1):795–804. doi: 10.1021/acs.biomac.5c02091 (PMC12806851; doi:10.1021/acs.biomac.5c02091)
Supplement: Supplementary file 1 [file bm5c02091_si_001.pdf]

Supporting information for:

Fish-based biopolymer complex coacervate coating for improved paper oxygen and water barrier

Sarah G. Fisher, Zachary Buck, Margaret J. Karim, Jaime C. Grunlan*

Texas A&M University, 400 Bizzell St., College Station, TX, 77840, USA

Corresponding author: Jaime C. Grunlan (jgrunlan@tamu.edu)

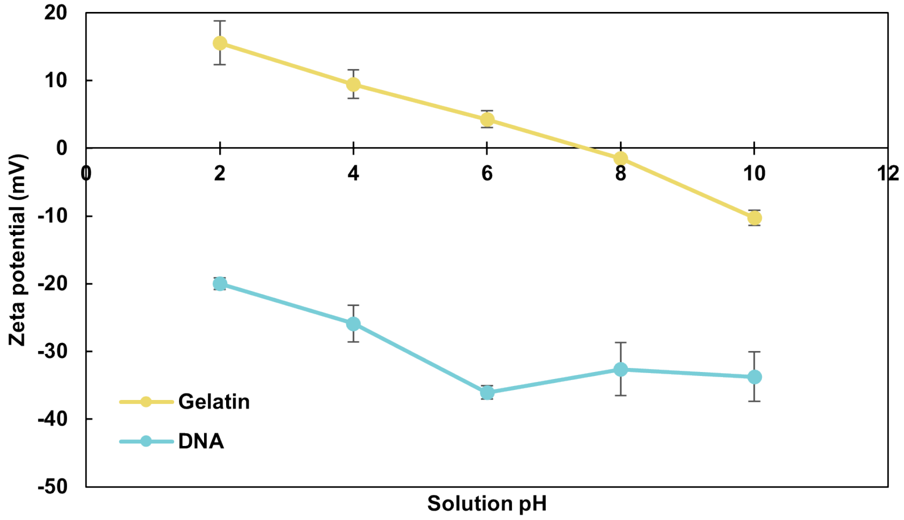

Figure S1. Zeta potential of gelatin and DNA solutions.

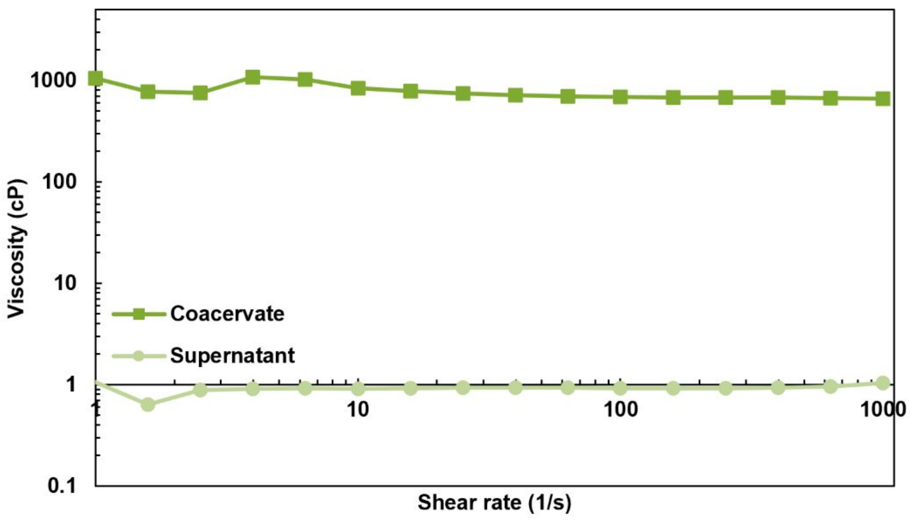

Figure S2. Rheology of coacervate and supernatant. Viscosity reported is an average of the datapoints from 100 to 100 1/s.

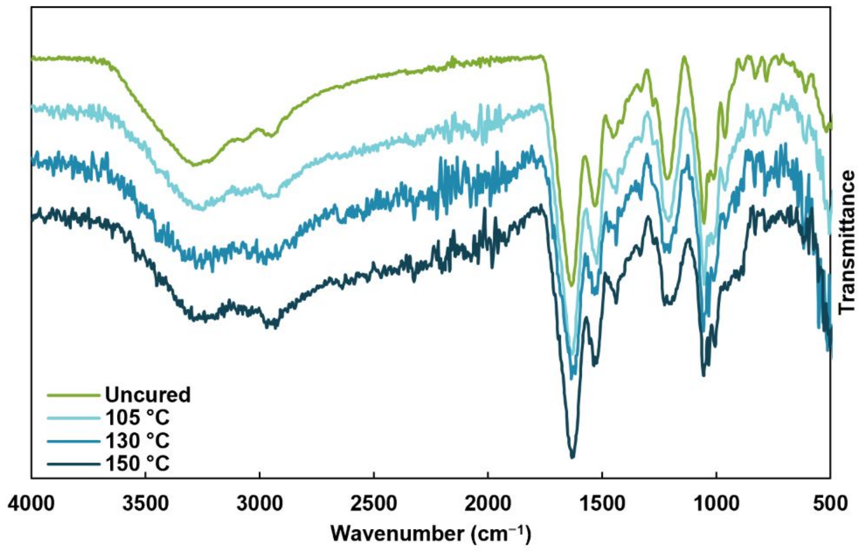

Figure S3. FTIR spectra of uncured and thermally cured coacervate coatings.

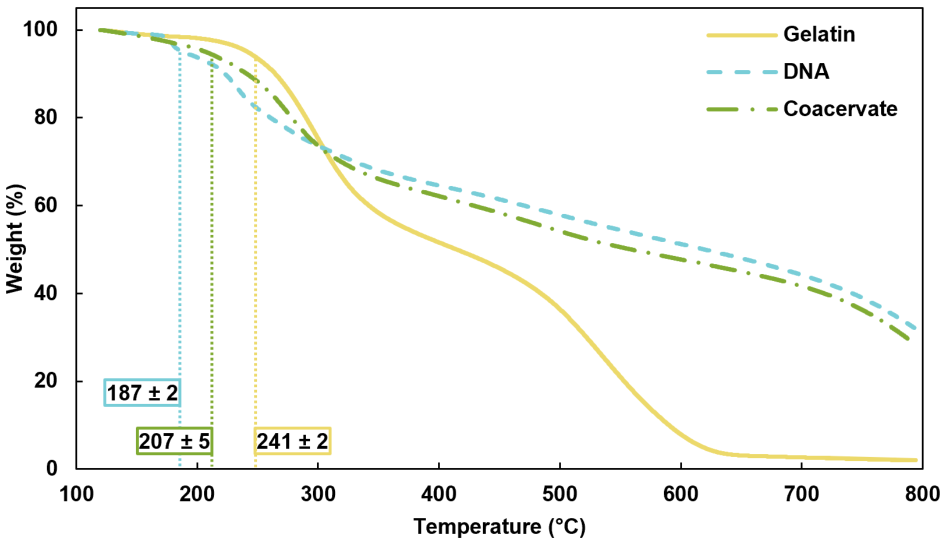

Figure S4. Representative thermal degradation curves of gelatin, DNA, and coacervate, with 5% degradation temperatures labeled.

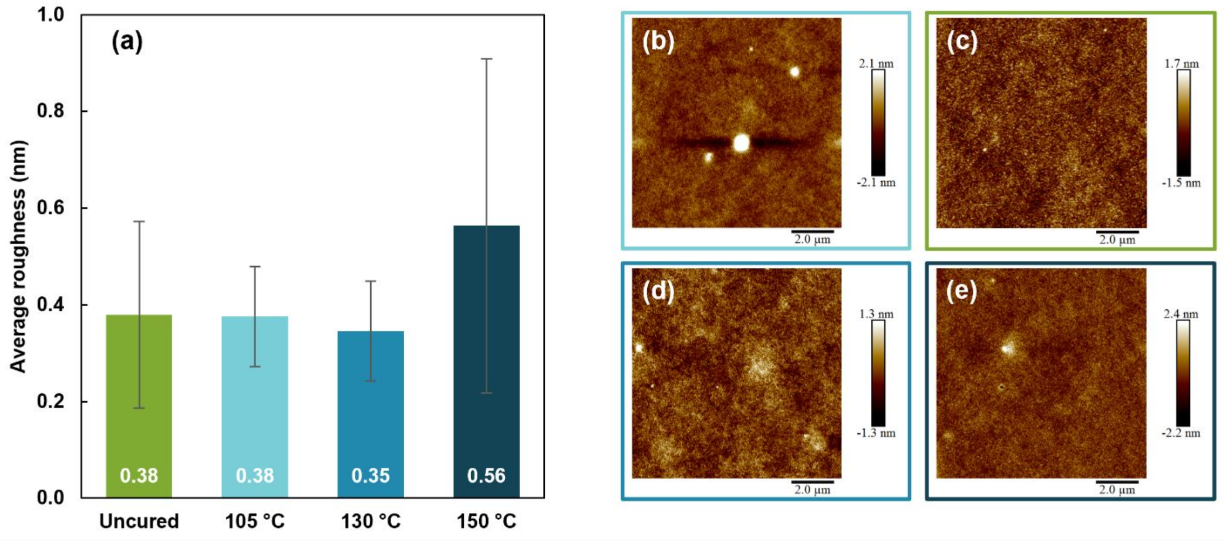

Figure S5. (a) Average roughness and (b–e) AFM images of coatings on silicon wafers: (b) uncured, (c) 105 ˚C, (d) 130 ˚C, (e) 150 ˚C.

Table S1. Relative atomic percentages and phosphorus-to-nitrogen ratios of gelatin, DNA, coacervate, and supernatant.

	Atom %					
	C	N	O	P	Na	P:N Ratio
Gelatin	64.6	17.3	18.0	—	—	0
DNA	61.4	12.1	20.5	4.9	1.0	0.41
Coacervate	58.9	20.4	18.0	2.7	—	0.13
Supernatant	50.2	21.1	20.0	6.6	21.1	0.31

Table S2. Expected gelatin to DNA molar ratios from measured phosphorus-to-nitrogen ratios.

	P:N Ratio	Mole fraction DNA	Gel:DNA Ratio
Pure gelatin	0	0	1:0
Pure DNA	0.41	1.01	0:1
Coacervate	0.13	0.40	1.5:1
Supernatant	0.31	0.82	0.22:1
Theoretical 1:1 molar ratio	*0.17*	*0.51*	*~1:1*

Mole fractions were calculated by the equation

$$P{:}N = \frac{4.9x}{12.1x + (1-x)17.3}$$

where x = the mole fraction of DNA in the sample. Gel:DNA ratios were calculated by the equation

$$Gel{:}DNA\ ratio = \frac{1-x}{x}$$
